# Elevated Levels of the Vesicular Monoamine Transporter and a Novel Repetitive Behavior in the *Drosophila* Model of Fragile X Syndrome

**DOI:** 10.1371/journal.pone.0027100

**Published:** 2011-11-02

**Authors:** John M. Tauber, Phillip A. Vanlandingham, Bing Zhang

**Affiliations:** Department of Zoology, University of Oklahoma, Norman, Oklahoma, United States of America; University of Missouri, United States of America

## Abstract

Fragile X Syndrome (FXS) is characterized by mental impairment and autism in humans, and it often features hyperactivity and repetitive behaviors. The mechanisms for the disease, however, remain poorly understood. Here we report that the *dfmr1* mutant in the *Drosophila* model of FXS grooms excessively, which may be regulated differentially by two signaling pathways. Blocking metabotropic glutamate receptor signaling enhances grooming in *dfmr1* mutant flies, whereas blocking the vesicular monoamine transporter (VMAT) suppresses excessive grooming. *dfmr1* mutant flies also exhibit elevated levels of VMAT mRNA and protein. These results suggest that enhanced monoamine signaling correlates with repetitive behaviors and hyperactivity associated with FXS.

## Introduction

Fragile X Syndrome (FXS) is the most common form of inheritable mental impairment and the leading identified cause of autism. It affects approximately 1/5000 males and roughly half as many females [1]. FXS is caused by the loss of the fragile X mental retardation protein (FMRP), largely due to transcriptional silencing that results from a tri-nucleotide (CGG) repeat expansion in the 5′ untranslated region of the fragile X mental retardation 1 (*FMR1*) gene [2]. In addition to cognitive impairment, individuals with FXS exhibit behavioral problems including hyperactivity, attentional deficits, and impulsivity [3], [4]. Autistic-like characteristics, such as anxiety and stereotypic, repetitive behavior, are also common features of FXS, and approximately 30% of patients meet the diagnostic criteria for autism [5], [6], [7], [8], [9]. Studying the role of FMRP in the nervous system is thus necessary to understand the pathogenesis of both mental impairment and autism and for developing new treatment strategies.

Mouse and fly models of FXS exhibit phenotypic defects remarkably similar to the human disorder. *Fmr1* knockout (KO) mice, which lack expression of the mouse homolog of FMRP, exhibit morphological abnormalities, learning and memory defects, and behavioral problems including attentional dysfunction, impulsivity, anxiety, and excessive grooming [10], [11], [12], [13]. The *Drosophila* gene *dfmr1* codes for the protein dFMRP [14], which contains the same functional domains as the mouse and human homologues [15]. *dfmr1* mutant flies exhibit defects in neuronal morphology [15], [16], [17], [18], physiology [17], [19], circadian rhythm [15], [20], [21], [22], sleep [23], courtship [20], learning, memory [24], and locomotion [17], [19], [25].

Although there is currently no effective treatment for FXS, research in the past decade has significantly advanced the understanding of the disorder. A major finding indicates that synaptic plasticity is altered in KO mice due to hyperactive signaling via the metabotropic glutamate receptor (mGluR) [26]. Further, reduction of mGluR expression in mutant mice, as well as treatment with mGluR antagonists, remarkably improves a number of phenotypes, including learning and memory [27], [28], [29]. However, these measures do not correct maroorchidism, indicating that enhanced mGluR signaling cannot account for all FXS phenotypes [28]. This partial rescue in mice is consistent with findings in *Drosophila* where reducing expression of DmGluR, the *Drosophila* homolog of mGluR, rescues neuronal overbranching [30] and physiological defects, but only partially improves synaptic physiology [31]. Further, blocking DmGluR signaling with the antagonist MPEP improves courtship, learning, memory, and rescues morphology defects in *dmfr1* mutant flies. MPEP, however, fails to rescue abnormal circadian rhythm and sleep [32]. These observations suggest that additional signaling pathways may be altered in both mouse and fly models of FXS. Given recent evidence suggesting that FMRP may regulate global translation, rather than just a subset of translation important for mGluR signaling, the inability of mGluR antagonists to correct all FXS defects is not surprising [33], [34]. While the evidence strongly suggests that cognitive impairment in FXS results from aberrant mGluR signaling, the neuronal mechanisms underlying hyperactivity, impulsivity, and autistic-like behaviors remain poorly understood.

Here we seek to determine other signaling pathways that may be affected in the absence of dFMRP. We first identify the novel behavioral phenotype of excessive grooming in *dfmr1* mutant flies, which appears to reflect the hyperactive and autistic-like features of FXS seen in mice and humans and adds another aspect of the disorder that can be studied in *Drosophila*. We find that blocking DmGluRs with MPEP does not reduce the excessive grooming in *dfmr1* mutant flies, supporting the idea that enhanced mGluR signaling underlies only a subset of FXS phenotypes. Instead, our results suggest that enhanced monoamine signaling correlates with the excessive repetitive behavior in *dfmr1* mutant flies.

## Results

### Wild-type *dfmr1* transgene rescues aberrant climbing in *dfmr1* mutant flies

*dfmr1* mutant flies are adult viable [17], but display a number of locomotion defects, including abnormal crawling as larvae [25] and impaired flight as adults [17]. We have shown that *dfmr1* mutant flies fail to climb robustly and that climbing progressively worsens with age [19]. To verify that abnormal climbing is directly caused by the loss of dFMRP, we introduced a transgene containing the wild-type *dfmr1* gene under the control of the endogenous promoter [20], into the *dfmr1* mutant background. We then investigated the climbing activity of genetically rescued mutant flies (hereafter called control), *dfmr1* mutant flies, and *dfmr1* mutant flies containing a *dfmr1* transgene with a frameshift (FS) in the open reading frame of the genomic rescue fragment (see Methods for further information on genotypes).

**Figure 1:**
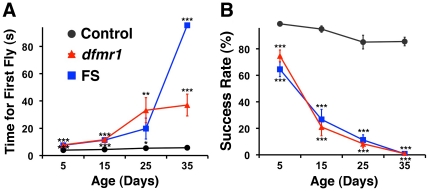
Aberrant climbing is rescued by genomic expression of the *dfrm1* gene. (**A**). The time for the first fly to climb 17.5 cm. *dfmr1* and FS (*dfmr1* with a wild-type *dfmr1* transgene that contains a frameshift mutation in the *dfmr1* open reading frame) do not express functional dFMRP, and show a progressive change in climbing behavior over the course of 35 days. The abnormal climbing is rescued by a transgene containing the genomic wild-type *dfmr1* locus (control). Data presented are the Mean +/- SEM (8 trials, total flies n = 80 for each genotype tested at each time point). (**B**). Total percentage of flies that successfully reach the 17.5 cm mark within 3 min. For all data, *p<0.05, **p<0.01, and ***p<0.001.

We monitored climbing performance at 5, 15, 25, and 35 days post-eclosion. We first measured the time for the first fly in a population of 10 flies to climb to a height of 17.5 cm (Movies S1 and S2). At 5 days old, the first fly in *dfrm1* and FS populations took, on average, 7.8 s and 7.5 s, respectively, to climb 17.5 cm, significantly longer than the average of 3.9 s for control flies (P<0.001; Fig. 1A). As the flies aged, the top performer in mutant populations took progressively longer to reach the target height (*dfmr1* averaged 37 s and FS averaged 96 s at 35 days old; Fig. 1A). In contrast, the climbing of top performers in control populations changed little with age, averaging 5.7 s at 35 days old.

To better reflect the climbing activity of all flies in a population, we counted how many flies reached 17.5 cm after 3 min and determined the success rate (Fig. 1B). Similar to the data for the top performers, 5 day-old mutant flies had a significantly lower success rate than controls (P<0.001; Fig. 1B), and their success rate declined with age. By 35 days, mutant groups approached a 0% success rate, a dramatic reduction from rates of 75% and 65% for 5 day-old *dfmr1* and FS flies, respectively (Fig. 1B). In contrast, control flies exhibited a noticeable, but much smaller, decline in success rate with age. We also measured the time for 50% of flies to reach 17.5 cm, as well as the failure rates for the first fly, or 50% of flies, in a population to complete the task successfully (Fig. S1). For all of these parameters we observed similar age-dependent declines in *dfmr1* mutant flies. Hence, our climbing data demonstrate an age-dependent decrease in climbing activity in *dfmr1* mutant flies, which is consistent with previous observations [19]. Further, adding a copy of the wild-type *dfmr1* gene to *dfmr1* mutant flies rescues the abnormal climbing behavior, indicating that this phenotype is caused specifically by the loss of dFMRP.

### *dfmr1* mutant flies groom excessively

**Figure 2:**
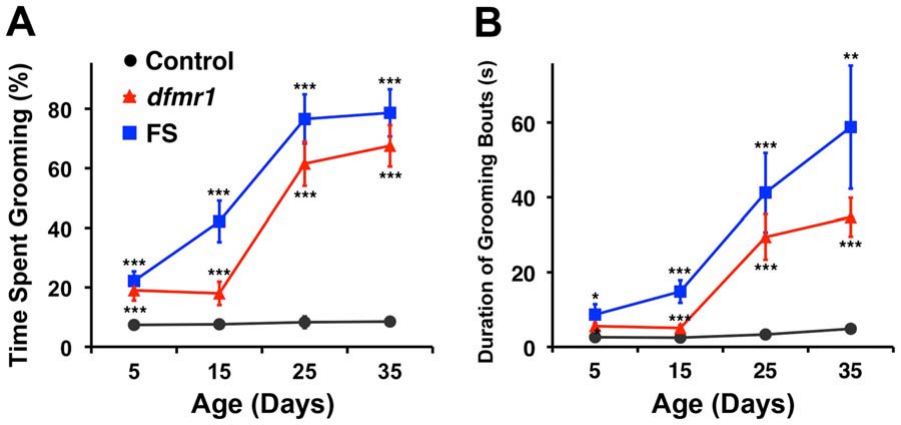
*dfmr1* mutant flies exhibit excessive grooming that increases with age. (**A**). At 5 days old, *dfmr1* and FS flies groom significantly more than control flies. Grooming increases with age in mutant flies; control flies show consistent levels of grooming at all ages tested. (**B**). The average duration of grooming bouts in mutant flies follows a similar trend to the total time spent grooming (A). In contrast, control flies show little change in the duration of grooming bouts from 5 to 35 days of age. Data are represented as the mean percentage of time single flies spend grooming during a 5 min period (Mean +/- SEM; n = 10–15 flies for each genotype at each time point). For all data, *p<0.05, **p<0.01, and ***p<0.001.

While performing the climbing tests we observed that *dfmr1* mutant flies frequently stopped climbing and began grooming themselves. To study this behavior more directly we recorded the activity of individual flies in a small observation chamber (see Methods; Movies S3 and S4). At 5 days old, *dfmr1* and FS flies groomed, on average, for 19% and 22% of the 5 min observation period, respectively (P<0.001; Fig. 2A). Control flies of the same age groomed significantly less, averaging 7% (Fig. 2A). Similar to the aberrant climbing, excessive grooming progressed with age in mutant flies, reaching as high as 79% in 35 day-old FS flies. Control flies, on average, spent 9% of the time grooming at 35 days, exhibiting little change in grooming activity with age.

In addition, the duration of individual grooming bouts increased in *dfmr1* mutant flies. At 5 days old, *dfmr1* and FS flies had slightly longer grooming bouts than control flies, but a significant difference occurred only for FS flies (*dfmr1*: P>0.05, FS: P<0.05; Fig. 2B). Similar to the overall grooming time in figure 2A, the average grooming bout duration increased with age in mutant flies (Fig. 2B). At 35 days, *dfmr1* and FS flies groomed 35 s and 59 s per bout on average, respectively, whereas control flies averaged 5 s per bout (*dfmr1*: P<0.001, FS: P<0.01; Fig. 2B).

### The mGluR antagonist MPEP partially rescues courtship behavior, but enhances excessive grooming

To gain insight into the mechanisms underlying the excessive grooming phenotype, we investigated the role of mGluR signaling. We raised larvae and maintained adult flies on food containing 86 µM MPEP, a dosage previously shown to rescue courtship, learning, memory, and neuronal morphology defects in *dfmr1* mutant flies [32]. We first tested the effects of MPEP on naïve courtship activity to ensure the drug's effectiveness. Courtship is an extensively studied, stereotypic behavior in *Drosophila* in which the male fly orients towards the female, tracks and follows her, produces wing songs, and attempts to lick her genitalia [35]. If the female is receptive, she then allows copulation. To quantify courtship activity, we used the standard ”Courtship Index” (CI), defined as the percentage of time a male fly spends performing any courtship behavior while in the presence of a female.

**Figure 3:**
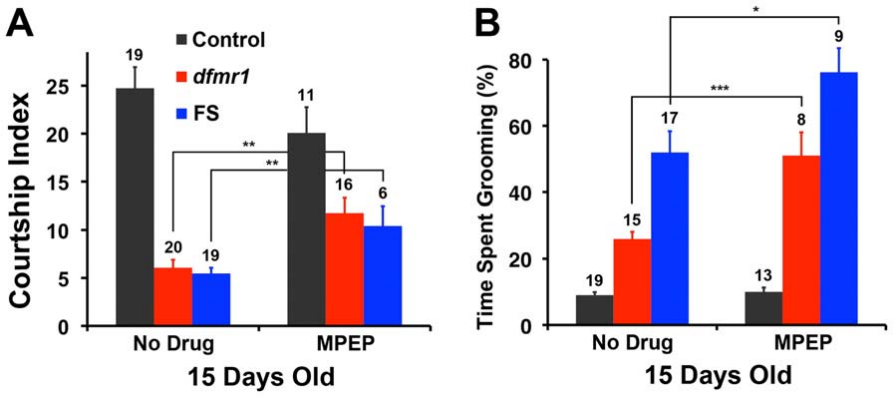
MPEP rescues courtship defects, but enhances excessive grooming in *dfmr1* mutant flies. (**A**). Treatment of *dfmr1* mutant male flies with MPEP improves courtship of naïve females. Flies were grown as larvae and maintained as adults on either control food or food containing 86 µM MPEP. Data are presented as the mean courtship index (CI, +/- SEM) with sample sizes shown above each bar. *dfmr1* and FS male flies treated with MPEP engage in courtship activity with wild-type virgin females significantly more than when treated with no drug. Control flies court less when treated with MPEP, but this difference is not significant. (**B**). 15 day-old *dfmr1* and FS flies treated with 86 µM MPEP groom significantly more than when treated with no drug. MPEP does not affect grooming activity in control flies. Data are presented as the mean percentage of time single flies spend grooming during a 5 min period (Mean +/- SEM); sample sizes are displayed above each bar. For all data, *p<0.05 and ***p<0.001 (Two-tailed students *t*-test).

In agreement with the results of previous studies [20], [32], we observed reduced naïve courtship in *dfmr1* mutant flies. Both 5 day-old *dfmr1* and FS flies had an average CI of approximately 6, whereas control flies had a markedly higher CI of 25 (Fig. 3A). Mutant flies treated with MPEP showed a partial, but significant, improvement in naïve courtship (P<0.01; Fig. 3A). Control flies exhibited a small decrease in CI when treated with the drug, but the change was not significant. Our results suggest that we administered MPEP properly.

We proceeded to test the effect of MPEP on excessive grooming. Surprisingly, we found that 15 day-old *dfmr1* and FS flies treated with MPEP showed a 2-fold and 1.5-fold increase in grooming activity, respectively, compared to those not treated with the drug (*dfmr1*: P<0.001, FS: P<0.05; Fig. 3B). In contrast, MPEP did not appear to affect the grooming activity of control flies (Fig. 3B).

**Figure 4:**
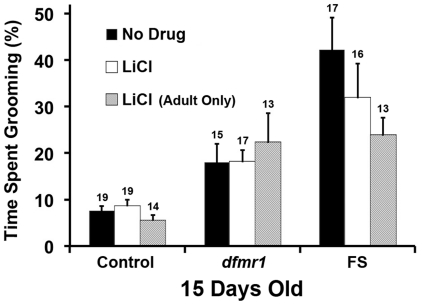
Lithium does not significantly affect grooming in *dfmr1* mutant flies. Grooming activity of 15 day-old flies grown as larvae and maintained as adults, or only maintained as adults on food containing 5 mM LiCl. Data are presented as the mean percentage of time single flies spend grooming during a 5 min period (Mean +/- SEM); sample sizes are displayed above each bar. For all genotypes, LiCl causes no significant change in grooming activity (Kruskal-Wallis one-way ANOVA with Dunn's post-hoc comparison).

In addition to MPEP, lithium (LiCl, 5 mM) has also been shown to rescue courtship, learning, and memory defects in *dfmr1* mutant flies [32]. In this study, 15 day-old *dfmr1* and FS flies grown as larvae and maintained as adults on food containing 5 mM lithium showed no significant changes in grooming activity, nor did control flies given the same treatment (Fig. 4). We also treated flies with lithium only as adults (i.e. larvae were grown on food with no drug), but found no difference in the drug's effect (Fig. 4). We therefore conclude that lithium, at a dosage that rescues other *dfmr1* behavioral defects, does not significantly affect the excessive grooming in *dfmr1* mutant flies.

### Reserpine suppresses excessive grooming in *dfmr1* mutant flies

As our results with MPEP suggested that excessive grooming in *dfmr1* mutant flies results from changes outside of mGluR signaling, we searched for other disturbances caused by the loss of dFMRP. FMRP has been shown to regulate dopamine signaling in both mouse and fly models of FXS. In cultured neurons of *Fmr1* KO mice, dopamine type 1 receptors are hyperphosphorylated and defective in signaling [36]. In *dfmr1* mutant flies, dopamine, and to a lesser extent serotonin, is elevated in the brain [37]. Moreover, biogenic monoamines have been shown to play a positive role in grooming in *Drosophila*. Application of dopamine, octopamine, and serotonin to the ventral nerve cord of decapitated flies stimulates grooming [38]. It has also been demonstrated that overexpression of the *Drosophila* vesicular monoamine transporter (dVMAT), which loads monoamines into synaptic vesicles, increases grooming in flies [39]. Finally, blocking dVMAT with the drug reserpine suppresses the elevated grooming activity in flies that overexpress the transporter [39]. Hence, we next tested the ability of reserpine to suppress excessive grooming in *dfmr1* mutant flies to examine if enhanced monoamine signaling might contribute to the behavior.

**Figure 5:**
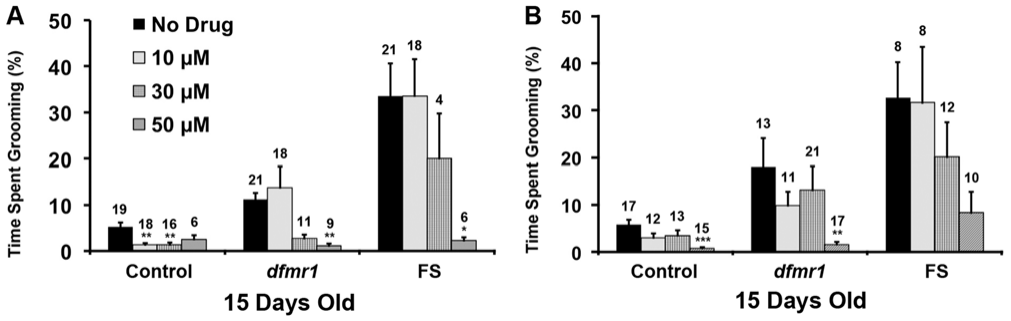
Dosage effects of reserpine on grooming. (**A**). Grooming activity of 15 day-old flies grown as larvae and maintained as adults on food containing no drug, 10, 30, or 50 µM reserpine (15 and 20 µM are omitted from figure). Reserpine suppresses grooming in *dfmr1* and FS flies, but only at 50 µM; control flies show significantly reduced grooming at the lowest concentration, 10 µM. (**B**). 15 day-old flies treated with reserpine only as adults (i.e. larvae were grown on control food), show a similar response. Suppressed grooming in mutant flies is only significant at 50 µM. In contrast, reserpine significantly decreases grooming activity in control flies at 15 µM. Data are presented as the mean percentage of time single flies spent grooming during a 5 min period (Mean +/- SEM); sample sizes are displayed above each bar. For all data, *p<0.05 and ***p<0.001 (Kruskal-Wallis one-way ANOVA with Dunn's post-hoc comparison).

15 day-old mutant flies treated with varying concentrations of reserpine (10, 15, 20, 30, and 50 µM) as both larvae and adults exhibited a significant decrease in grooming at 50 µM (*dfmr1*: P<0.01, FS: P<0.05), but not at the lower concentrations (Fig. 5A). In contrast, control flies treated with reserpine as larvae and adults showed significantly suppressed grooming at 10 µM (P<0.01), 20 µM (P<0.001), and 30 µM (P<0.01) (Fig. 5A). To determine if reserpine is effective post-developmentally, we treated flies with the drug only after eclosion. Similar to figure 5A, 15 day-old *dfmr1* flies treated only as adults groomed significantly less at 50 µM (P<0.01), but not at lower concentrations (Fig. 5B). FS flies treated with reserpine only as adults did not show a significant difference in grooming at any concentration, but it is worth noting that the reduction of grooming activity for 50 µM became significant upon exclusion of an outlier in the sample (data not shown). Like in figure 5A, post-developmental reserpine treatment significantly reduced grooming in controls at lower dosages than in mutants (Fig. 5B). Our results demonstrate that reserpine can effectively suppress excessive grooming in *dfmr1* mutant flies, is effective when used only in adulthood, and that *dfmr1* mutant flies are less sensitive to reserpine than control flies.

### *dVMAT* mRNA and protein levels are elevated in the absence of dFMRP

**Figure 6:**
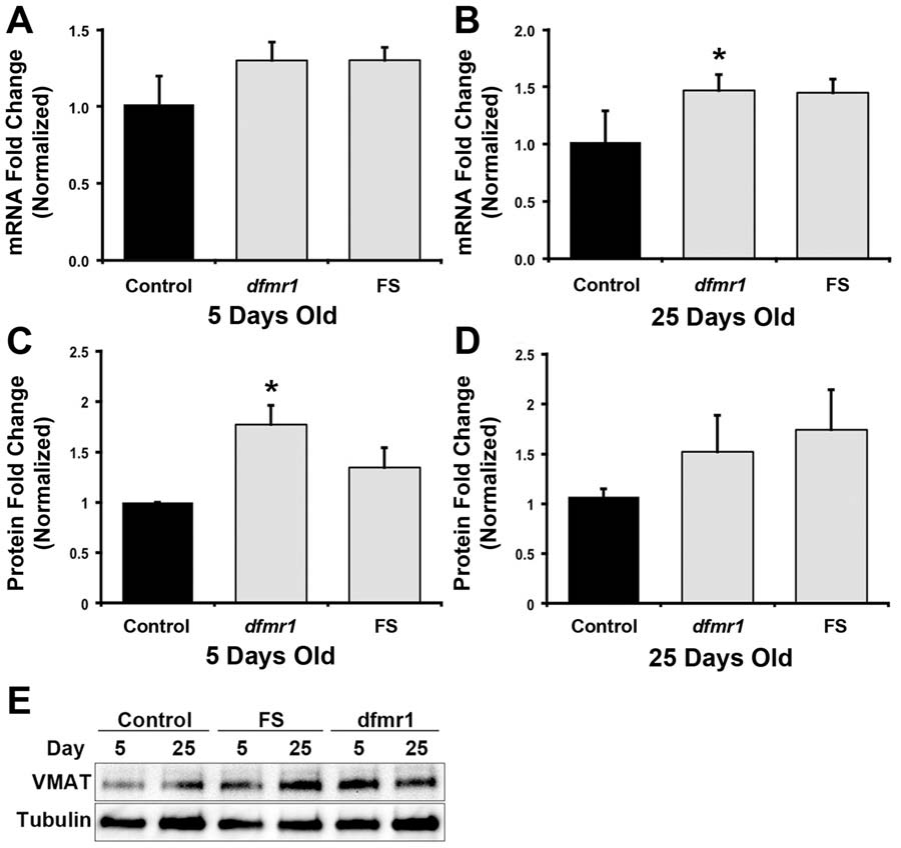
*dVMAT* transcript and protein levels are upregulated in *dfmr1* mutant flies. Quantitative real-time PCR experiments indicate that *dVMAT* mRNA levels are increased in 5 day-old *dfmr1* mutant flies (**A**) and in 25 day-old *dfmr1* mutant flies (**B**) relative to control flies. Western blot analyses indicate that *dfmr1* mutant flies also have increased levels of dVMAT protein at both (**C**) 5 days and (**D**) 25 days of age. (**E**) Representative blot showing increased dVMAT protein levels in *dfmr1* and FS flies compared to control flies. Real-time PCR data are presented as the average of three biological replicates. Western blot data are shown as the average of three independent trials. *p<0.05 (One-way ANOVA with Dunnett's post-hoc comparison).

As suppression of excessive grooming in mutant flies required a higher dosage of reserpine, we hypothesized that dVMAT levels could be increased in *dfmr1* mutant flies. Although dFMRP is primarily known to regulate translation, it has also been shown to influence transcript expression [40], [41], [42], [43]. To determine if *dVMAT* mRNA levels are affected by the loss of dFMRP, we used quantitative real-time polymerase chain reaction (qPCR) to quantify *dVMAT* transcript levels in *dfmr1* mutant flies. At 5 days of age, *dfmr1* mutant flies showed a 29% increase in *dVMAT* mRNA compared to control flies, but the change was not significant (P>0.05; Fig. 6A). At 25 days of age, we detected a 41% increase in *dVMAT* transcript levels in mutant flies compared to control flies. This increase was significant for *dfmr1* flies, but not for FS flies (*dfmr1*: P<0.05, FS: P>0.05; Fig. 6B). Thus, *dVMAT* transcript levels increased in *dfmr1* mutant flies over time, up to the age we tested.

We next examined the effect of the absence of dFMRP on dVMAT protein levels. Consistent with the increase in mRNA, we detected a 77% and 35% elevation of dVMAT protein levels in 5 day-old *dfmr1* and FS fly heads, respectively, compared to 5 day-old control fly heads. This represents a significant increase in dVMAT protein levels for *dfmr1* but not for FS flies (*dfmr1*: P<0.05; FS: P>0.05; Fig. 6C). We also detected elevated dVMAT protein levels at 25 days in *dfmr1* (52%) and FS (74%) flies, but neither increase was statistically significant compared to control flies at both 5 and 25 days (Fig. 6D). Hence, our results show a trend that dVMAT transcript and protein levels are both upregulated in *dfmr1* mutant flies, and under certain conditions the increases reach statistically significant levels.

## Discussion

There are four major findings from this study: 1) age-dependent abnormal climbing in *dfmr1* mutant flies can be genetically rescued, 2) excessive grooming is identified as a new behavioral defect in *dfmr1* mutant flies, 3) excessive grooming can be suppressed by reserpine, and 4) *dVMAT* mRNA and protein levels are increased in the absence of dFMRP.

In a previous study we revealed abnormal climbing activity in *dfmr1* mutant flies that progresses with age. Our results in this study confirm this finding, and additionally show that introducing a wild-type *dfmr1* transgene into the *dfmr1* mutant background restores normal climbing behavior. Further, a frameshift mutation in the open reading frame of the transgene abolishes the rescue of climbing behavior. These results demonstrate that the abnormal climbing in *dfmr1* mutant flies is directly caused by the loss of dFMRP.

In this study we have also identified excessive grooming as an important and novel behavioral defect in the fly model of FXS. Our results show that *dfmr1* mutant flies groom significantly more than control flies, and that mutant flies also have significantly longer grooming bouts. Further, this excessive grooming intensifies with age in *dfmr1* mutant flies, whereas control flies show essentially no change in grooming activity over time. A wild-type copy of the *dfmr1* gene can rescue the excessive grooming defect in *dfmr1* mutant flies. It is worth noting that FS mutants show more dramatic climbing and grooming defects compared to *dfmr1* mutants. We do not know the exact underlying cause, but it may be due to the presence of either mRNA or a truncated peptide produced from the FS rescue fragment, having gain of function effects.

In video recordings, control flies mostly walk around the observation chamber and groom occasionally, but rarely stand motionless. In contrast, the mutant fly spends more time grooming. It is possible that grooming is a default activity that occurs whenever a fly is not walking. If this is the case, excessive grooming in *dfmr1* mutant flies could result indirectly from problems in walking. This explanation would be consistent with our observation that *dfmr1* mutant flies exhibit postural problems and uncoordinated movement. However, the *dfmr1* flies are capable of climbing after a brief period of mechanical disturbance (i.e., knocking them down in the graduated cylinder), albeit at a slower speed compared to control flies. Further, reserpine suppresses grooming in *dfmr1* mutant flies without improving walking (data not shown). These observations suggest that grooming is not simply a default behavior in the absence of walking and that *dfmr1* mutations specifically cause excessive grooming. Notably, *Fmr1* KO mice have also been reported to exhibit excessive grooming when presented with social stimuli [11], [13]. A study of self-injurious behavior in FXS patients reported a prevalence of harmful rubs and scratches [44]. Hence, heightened repetitive activity such as grooming is a common behavioral defect in FXS.

Although reducing mGluR signaling has been shown to rescue learning and memory defects in both mouse and fly FXS models, we find that the mGluR antagonist MPEP enhances excessive grooming in *dfmr1* mutant flies. This is not completely surprising, as the absence of dFMRP likely alters numerous signaling pathways and developmental processes of the nervous system. MPEP also fails to rescue abnormal sleep [23] and circadian rhythm [32] in *dfmr1* mutant flies, which may impact locomotor activity like grooming. It is worth noting that *dfmr1* mutant flies did not groom more when treated with LiCl, suggesting that mGluR antagonists and LiCl may have different neuronal targets. An interesting question that arises from these results is whether an mGluR agonist might suppress grooming in *dfmr1* mutant flies. Previous results have shown that glutamate at concentrations as low as 5 µM is toxic to *dfmr1* mutant flies and significantly affects various behaviors in the fly [45]. This makes it difficult to assess the potential benefit of mGluR agonists on grooming.

Previous work shows that dopamine plays a role in FXS in both mice [36] and *Drosophila* [37], and that biogenic monoamines stimulate fly grooming [38], [39]. In our studies, blocking dVMAT with reserpine suppresses excessive grooming in *dfmr1* mutant flies, but only significantly at 50 µM. Control flies groom significantly less when treated with just 10 µM. These results indicate that *dfmr1* mutant flies are less sensitive to reserpine's effect on grooming. However, we cannot exclude the possibility that reserpine has additional targets and therefore generally sedates the fly. Both suppression of dVMAT as well as a non-specific target could slow down most motor activities including grooming. Alternatively, it is possible that basal monoamine activity is required for grooming, and therefore shutting down monoamine signaling may block the behavior.

In our study, we find elevated levels of *dVMAT* transcript and protein in *dfmr1* mutant flies. Although these increases are not statistically significant in some instances, they are consistent in both mutant lines. However, it is not clear from our results how the loss of dFMRP leads to increased *dVMAT* expression. The transcription of *dVMAT* may be directly increased. Alternatively, degradation of *dVMAT* mRNA may decrease in the absence of dFMRP, a distinct possibility as FMRP has been previously indicated to regulate mRNA stability [46]. How dFMRP regulates dVMAT protein levels is also unclear. Elevated dVMAT protein levels may occur exclusively because of increased transcript levels, but could also result from increased translation or reduced degradation of the protein. Nonetheless, our observations are in agreement with the known function of FMRP as a regulator of transcription and translation [40], [42], [47].

Many factors may contribute to the excessive grooming in *dfmr1* mutant flies, and our data do not resolve whether upregulation of dVMAT directly influences this behavior. Overexpression of dVMAT stimulates grooming in flies [39], and dopamine levels are increased in *dfmr1* mutant brains [37]. Monoamines could directly or indirectly modulate multiple downstream signaling pathways involved in grooming. The hyposensitivity to reserpine seems to suggest that a greater number of dVMATs are present on mutant synaptic vesicles, as a higher concentration of the drug is required to reduce grooming. We note that overexpression of dVMAT in serotonergic and dopaminergic neurons leads to hypersensitivity to reserpine on grooming [39]. One likely explanation of these differences is that *dfmr1* mutations affect not only monoamine cells but also other cells such as neurons in the mushroom bodies and neurons postsynaptic to monoamine cells. We are also are aware that dopamine signaling is reduced in the forebrain of *Fmr1* KO mice [36]. Thus, while plausible given the effect of reserpine, we cannot establish a clear causal relationship between excessive grooming and dVMAT expression levels.

Understanding how FMRP functions in development and aging will be crucial for effective treatment of FXS [48], [49]. Studies in mouse and *Drosophila* indicate that FMRP is temporally regulated and that treatment requires proper timing [50], [51], [52], [53], [54]. Our results add to the growing evidence of the importance of FMRP in age-related processes, and also demonstrate that hyperactivity and repetitive behavior increase with age in the *Drosophila* model. Interestingly, the severity of autistic behavior and anxiety has been found to increase with age in studies of FXS patients [55], [56]. Our results indicating that reserpine is effective in adult *dfmr1* mutant flies could help develop or improve treatment, as they suggest that hyperactive and repetitive behavior in older patients is potentially reversible.

Although we believe excessive grooming in *dfmr1* mutant flies is a model of an impulsive and repetitive behavior, animal models can never completely recapitulate human disorders. The mechanisms underlying repetitive behaviors in FXS patients are likely much more complex. Nonetheless, we demonstrate a correlation between monoamine signaling and the excessive grooming phenotype in *dfmr1* mutant flies and that VMAT is a protein that merits further study in FXS. Importantly, our study provides potentially useful information for improving the pharmaceutical treatment of FXS symptoms in human patients.

## Materials and Methods

### Fly stocks, Genetics, and Pharmacology

Flies were grown on a standard cornmeal-agar medium under a 12 h/12 h light/dark cycle. *fragile X* (*dfmr1*) mutant flies were generated by crossing *w; dfmr1^83M^/TM6B, Tb* with *w; dfmr1^3^/TM6C, Sb* flies and selecting *w; dfmr1^83M^/dfmr1^3^* flies from the progeny [17], [20]. Control flies, which contain a transgene encoding the wild-type *dfmr1* gene in the *dfmr1* mutant background, were generated by crossing *w; dfmr1^83M^/TM6B, Tb* with *w; wild-type rescue (WT)/+; dfmr1^3^/TM6C, Sb* flies [20]. FS flies, which carry a frameshift in the open reading frame of the transgenic rescue fragment were generated by crossing *w; dfmr1^83M^/TM6B, Tb with w; frameshift rescue (FS)/+; dfmr1^3^/TM6C, Sb* flies [20]. For grooming assays, 1–3 day-old adult male flies were collected following brief anesthetization with CO_2_. Flies were stored in fresh food vials with 10–13 flies per vial (climbing) or 2–8 flies per vial (grooming). For MPEP and LiCl administration, an aqueous stock solution was mixed into recently cooked standard food after the food had cooled. As reserpine is insoluble in water, a stock solution in 1 M acetic acid was mixed into molten food in a 1∶9 ratio [39]. For control experiments, flies were raised on food containing the same amount of each vehicle (water or acetic acid). Flies were transferred to new food vials every 4–6 days.

### Behavioral Assays

#### Climbing

For climbing trials, 10 male flies were transferred to a 250 mL glass graduated cylinder, which was sealed with parafilm to prevent escape. Next, the flies were knocked down to the bottom; and care was taken to use similar force for all trials. Measurements were taken for the (1) time for the first fly to cross the 150 mL line (17.5 cm from the bottom); (2) percentage of trials when a first fly did not cross the 17.5 cm line within 3 min; (3) time for 50% of the population to cross the 17.5 cm line; (4) percentage of trials when 50% of the population did not cross the 17.5 cm line within 3 min; and (5) the percentage of flies that crossed the 17.5 cm line within 3 min. Four trials were performed for each population and their average was taken for a sample value. A total of 8 samples were taken for each genotype. For data analysis we excluded events for (1) in which no fly, and for (3) in which 50% of a population, did not reach 17.5 cm within 3 min. Experiments were performed between 5–7 pm to minimize potential effects of circadian oscillation.

#### Courtship

Virgin male flies collected within 4 hours of eclosion were stored in individual food vials. Virgin wild-type (CS, Canton S) female flies, collected on the same day as the virgin males, were kept in groups of 10–20 per food vial. All courtship assays were performed with 5 day-old male and female flies between 3–6 pm. Male flies were first aspirated into an observation chamber of about 0.4 cm^3^, and after 1 min of acclimation, a virgin wild-type female was aspirated into the chamber and behavior was then monitored for 10 min. The courtship index (CI) was scored as the percentage of time that a male fly spent engaged in courtship activity while paired with a female [32], [57]. Courtship behavior was recorded on video and analyzed later using the iVideo program for Macintosh.

#### Grooming

Single male flies were aspirated into a 0.4 cm^3^ observation chamber, allowed to acclimate for 1 min, and then recorded for a 5 min observation period. Data were collected for (1) the percentage of time the fly spent grooming and (2) the duration of individual grooming bouts. Grooming bouts were recorded as ending when a fly either stopped grooming and remained motionless for 2 s, or stopped grooming and walked at least 4 steps. Grooming experiments were performed between 3–6 pm and were recorded and analyzed using video software.

### Quantitative Real-time PCR

Flies were collected within 24 hours of eclosion, aged for 5 days or 25 days, and frozen in liquid nitrogen between 3–6 pm. Samples were stored at −80°C. Three biological replicates were used for each genotype at each age. RNA was extracted from 40 flies with TRIzol (Invitrogen) and purified using a Qiagen RNeasy Mini Kit (Qiagen) with on-column DNase I (Qiagen) treatment. RNA yield and purity was checked with a NanoDrop 2000 Specrophotometer (Thermo Scientific). To generate cDNA, 0.4 µg of RNA was used with a SuperScript III Reverse Transcriptase First-Strand Synthesis Kit (Invitrogen). Real-time PCR was performed using a Maxima SYBR Green/ROX qPCR Master Mix (Fermentas). Analyses were performed using an Applied Biosystems 7500 Real-time PCR System. Relative expression levels were determined with the 2^−ddCt^ method [58], using *rp49* as a reference gene. qPCR primer sequences for *dVMAT* were:

5′-AAAATTGGACGATGGTTTGC-3′ (forward) and

5′-ATTCGGGATGATCAGGTGAG-3′ (reverse);

primer sequences for *rp49* were:

5′-CGGATCGATATGCTAAGCTGT-3′ (forward) and

5′-GCGCTTGTTCGATCCGTA-3′ (reverse).

### Western blots

Flies were collected within 24 hours of eclosion, aged for 5 days or 25 days, and frozen in liquid nitrogen between 3–6 pm. Samples were stored at −80°C. An equal number of fly heads were isolated for each experiment (15–20 total/condition), homogenized in Buffer A (150 mM NaCl, 10 mM HEPES, pH 7.4, 1 mM EGTA, 0.1 mM MgCl_2_, 2 mM PMSF, and protease inhibitor cocktail) (Roche, Indianapolis, IN) using a plastic pestle. Protein lysates were cleared by centrifugation at 22,000 x g for 20 min. Total protein concentration was measured by a BCA Assay (Thermo Scientific, Rockford, IL), diluted with 2 x SDS Sample Buffer, boiled for 5 min, and equal concentrations of protein were separated on a 10% SDS-PAGE gel for each condition. Following transfer to nitrocellulose, the membranes were incubated overnight at 4°C at 1∶4000 in rabbit anti-dVMAT [59], washed in 0.5% TBS-Tween, and incubated for 1 h at 1∶4000 in HRP-conjugated rabbit secondary antibody. Protein was detected using the ECL method, normalized to tubulin (Sigma, St. Louis, MO), and quantified using Image J (NIH).

### Statistical Analysis

Most statistical analyses were performed using GraphPad Prism 4. ANOVA with a Dunnett's multiple comparison test was used for statistical analysis, unless noted otherwise. For data that were not normally distributed, and for which transformation could not resolve this issue, a non-parametric test was used (Kruskal-Wallis one-way ANOVA with a Dunn's post-hoc test). All data are shown as mean +/- SEM. *p<0.05, **p<0.01, and ***p<0.001 are considered statistically significant.

## Supporting Information

Figure S1 **Additional measurements of climbing behavior in** ***dfmr1*** **mutant flies.** (**A**). Time for 50% of a population to climb 17.5 cm. Control flies contain a wild-type *dfmr1* transgene under endogenous regulation in the *dfmr1* mutant background. *dfmr1* and FS (*dfmr1* mutants that contain a wild-type *dfmr1* transgene that has a frameshift mutation in the *dfmr1* open reading frame) do not express functional dFMRP. By 35 days all *dfmr1* and FS populations failed to have 50% reach 17.5 cm within 3 min. Data presented are the average of Mean +/- SEM (8 trials, total flies n = 80 for each genotype tested at each time point). (**B**). Percentage of failed attempts for populations to have a first fly reaching the 17.5 cm line. (**C**). Percentage of failed attempts for populations to have at least 50% of flies climb 17.5 cm. For all data, *p<0.05, **p<0.01, and ***p<0.001.
(TIF)

Movie S1 Sample video of control flies climbing. This video illustrates how climbing experiments were conducted. Ten control flies at 15 day-old were gently knocked to the bottom of a graduated cylinder and then observed to climb to the top.
(MOV)

Movie S2 Sample video of the *dfmr1* mutant flies climbing. Ten *dfmr1* mutant flies at 15 day-old were gently knocked to the bottom of a graduated cylinder. The flies then begin climbing, but some stop after a short period of time. Later analysis showed that these flies stopped to groom themselves.
(MOV)

Movie S3 Sample video of *dfmr1* mutant grooming activity. A 15 day-old *dfmr1* mutant fly initially explores the environment for 10 s, but then begins grooming excessively.
(MOV)

Movie S4 Sample video of grooming activity in a control fly. A 15 day-old control fly explores the environment, stopping only once to groom for 3 s.
(MOV)
