# Supplementary figures and images for: Elevated Levels of the Vesicular Monoamine Transporter and a Novel Repetitive Behavior in the Drosophila Model of Fragile X Syndrome
Source: PLoS One. 2011 Nov 2;6(11):e27100. doi: 10.1371/journal.pone.0027100 (PMC3206932; doi:10.1371/journal.pone.0027100)

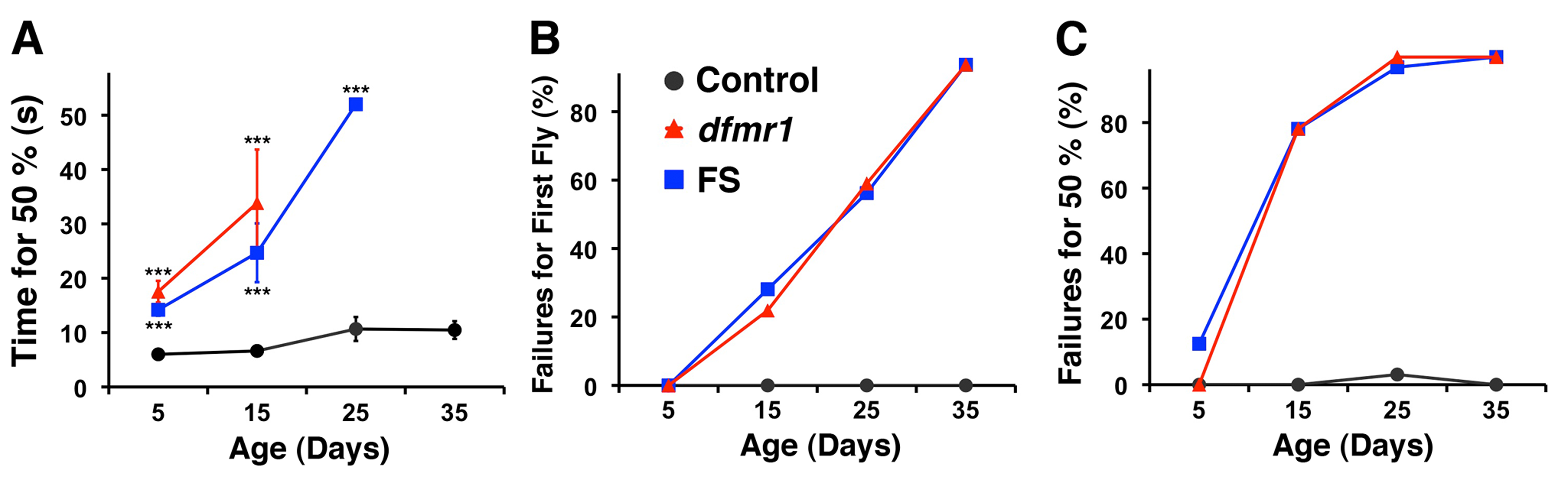

Supplement: Figure S1 — Additional measurements of climbing behavior in dfmr1 mutant flies. (A). Time for 50% of a population to climb 17.5 cm. Control flies contain a wild-type dfmr1 transgene under endogenous regulation in the dfmr1 mutant background. dfmr1 and FS (dfmr1 mutants that contain a wild-type dfmr1 transgene that has a frameshift mutation in the dfmr1 open reading frame) do not express functional dFMRP. By 35 days all dfmr1 and FS populations failed to have 50% reach 17.5 cm within 3 min. Data presented are the average of Mean +/- SEM (8 trials, total flies n = 80 for each genotype tested at each time point). (B). Percentage of failed attempts for populations to have a first fly reaching the 17.5 cm line. (C). Percentage of failed attempts for populations to have at least 50% of flies climb 17.5 cm. For all data, *p<0.05, **p<0.01, and ***p<0.001. (TIF) [file pone.0027100.s001.tif]
